# A Low Tacrolimus Concentration/Dose Ratio Increases the Risk for the Development of Acute Calcineurin Inhibitor-Induced Nephrotoxicity

**DOI:** 10.3390/jcm8101586

**Published:** 2019-10-02

**Authors:** Gerold Thölking, Katharina Schütte-Nütgen, Julia Schmitz, Alexandros Rovas, Maximilian Dahmen, Joachim Bautz, Ulrich Jehn, Hermann Pavenstädt, Barbara Heitplatz, Veerle Van Marck, Barbara Suwelack, Stefan Reuter

**Affiliations:** 1 Department of Medicine D, Division of General Internal Medicine, Nephrology and Rheumatology, University Hospital of Münster, 48149 Münster, Germany; Katharina.schuette-nuetgen@ukmuenster.de (K.S.-N.); schmitzjulia.js@googlemail.com (J.S.); Alexandros.rovas@ukmuenster.de (A.R.); Maximilian.dahmen@ukmuenster.de (M.D.); joachim.bautz@ukmuenster.de (J.B.); ulrich.jehn@ukmuenster.de (U.J.); herman.pavenstaedt@ukmuenster.de (H.P.); Barbara.Suwelack@ukmuenster.de (B.S.); 2 Department of Internal Medicine and Nephrology, University Hospital of Münster, Marienhospital Steinfurt, 48565 Steinfurt, Germany; 3 Gerhard-Domagk-Institute of Pathology, University Hospital of Münster, 48149 Münster, Germany; barbara.heitplatz@ukmuenster.de (B.H.); veerle.vanmarck@ukmuenster.de (V.V.M.)

**Keywords:** calcineurin inhibitor nephrotoxcity, tacrolimus, C/D ratio, tacrolimus metabolism, kidney transplantation

## Abstract

Fast tacrolimus metabolism is linked to inferior outcomes such as rejection and lower renal function after kidney transplantation. Renal calcineurin-inhibitor toxicity is a common adverse effect of tacrolimus therapy. The present contribution hypothesized that tacrolimus-induced nephrotoxicity is related to a low concentration/dose (C/D) ratio. We analyzed renal tubular epithelial cell cultures and 55 consecutive kidney transplant biopsy samples with tacrolimus-induced toxicity, the C/D ratio, C0, C2, and C4 Tac levels, pulse wave velocity analyses, and sublingual endothelial glycocalyx dimensions in the selected kidney transplant patients. A low C/D ratio (C/D ratio < 1.05 ng/mL×1/mg) was linked with higher C2 tacrolimus blood concentrations (19.2 ± 8.7 µg/L vs. 12.2 ± 5.2 µg/L respectively; *p* = 0.001) and higher degrees of nephrotoxicity despite comparable trough levels (6.3 ± 2.4 µg/L vs. 6.6 ± 2.2 µg/L respectively; *p* = 0.669). However, the tacrolimus metabolism rate did not affect the pulse wave velocity or glycocalyx in patients. In renal tubular epithelial cells exposed to tacrolimus according to a fast metabolism pharmacokinetic profile it led to reduced viability and increased Fn14 expression. We conclude from our data that the C/D ratio may be an appropriate tool for identifying patients at risk of developing calcineurin-inhibitor toxicity.

## 1. Introduction

Although the calcineurin inhibitor (CNI) tacrolimus (Tac) is effective in preventing graft rejection after transplantation, its therapeutic window is narrow. Furthermore, Tac exhibits a high intra- and inter-individual variability in pharmacokinetics (PK) and pharmacodynamics [1]. Tac-related adverse effects are common even in patients with Tac trough levels within the intended therapeutic range, despite meticulous therapeutic drug monitoring. CNI-induced nephrotoxicity (CNIT) especially remains a severe issue during CNI treatment [2]. While acute CNIT comprises isometric tubular vacuolization, acute arteriolopathy, and thrombotic microangiopathy, the features of chronic CNIT include interstitial fibrosis and tubular atrophy, arteriolar hyalinosis, tubular microcalcifications, and global glomerulosclerosis [2]. Unfortunately, there are no specific molecular markers of CNIT, but it was recently experimentally shown that, e.g., the TWEAK/Fn14 pathway, is critically involved in the pathogenesis of CNIT [3]. Although it is known that overexposure to Tac causes CNIT trough level-dependently, even patients presenting with Tac trough levels within the therapeutic range (5–15 µg/L) are vulnerable to developing both acute or chronic CNIT [4,5,6,7,8,9]. This indicates the possibility of additional causative factors.

Using the concentration/dose (C/D) ratio, a strong association between a fast Tac metabolism rate/fast oral Tac clearance (C/D ratio < 1.05 µg/L×1/mg) and reduced renal function within the first month following renal transplantation (RTx) can be demonstrated [9]. Other studies found comparable outcomes; even after liver transplantation [7,10,11,12,13,14]. However, this effect cannot be observed, if considerably higher C/D-ratio cut-offs are chosen [15]. The Tac metabolism effect on renal function was detectable even five years after RTx and was also associated with increased mortality in patients with a low C/D ratio [16]. 

What are the reasons for these findings? Apart from an increased susceptibility of fast metabolizers to BK virus infections, these patients more frequently required indication biopsies that revealed higher rates of rejections and acute CNIT [9,16,17]. Therefore, we hypothesized that the C/D ratio as a simple estimate of the Tac metabolism correlates with the severity of CNIT.

## 2. Experimental Section

### 2.1. Patients and Histology

At first, the study was conducted to answer the question if there is an association between histological findings of acute CNIT and the corresponding Tac C/D ratio at the time of biopsy. We hypothesized that a C/D ratio <1.05 ng/mL×1/mg is associated with acute CNIT. To prove the hypothesis, we performed a histological reevaluation of all for-cause RTx-biopsy samples that showed acute CNIT in our center between 2007 and 2016. Only samples with definite histological signs of acute CNIT (isometric vacuolization of tubular epithelial cells) were included in the study. Biopsy samples with isometric vacuolization that could be attributed to other causes were excluded from the evaluation.

**Figure 1:**
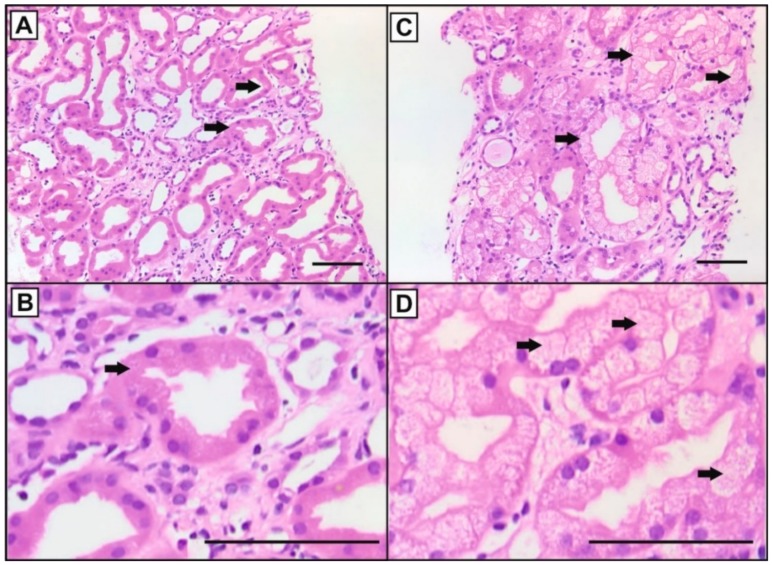
Examples of Hematoxylin and Eosin (HE)-stained sections of kidney transplant biopsies with different overall scores of isometric vacuolization (arrows) as a marker of calcineurin inhibitor-induced nephrotoxicity. (**A**): < 25% of the tubular epithelial cells, (**B**): magnification of A, (**C**) ≥ 25% of the tubular epithelial cells, and (**D**): magnification of C (bars: 100 µm).

Two pathologists, independently and blinded, categorized the graft biopsies in the following groups: <10%, 10–25%, 25–50%, and ≥50% of tubules showing isometric vacuolization of the cytoplasm (Figure 1A–D). In case of different assessment of the pathologists, mean values were taken. Due to limited sample numbers, for final analysis two categories were considered: samples with <25% and with ≥25% affected tubular cells (*n* = 35 and *n* = 20, respectively).

The C/D ratio was calculated by the Tac blood trough concentrations and the corresponding Tac doses on the day of the renal biopsy. C/D ratio values < 1.05 ng/mL×1/mg defined patients as fast Tac metabolizers (patients with fast oral clearance), values ≥ 1.05 ng/mL×1/mg characterized slow metabolizers (patients with slow oral clearance) as published before [8,13]. Only 12 h Tac trough levels were used for this analysis.

After confirmation of our first hypothesis, we secondly designed a prospective part of the study to address the question, if CNIT could be related to Tac peak levels. We hypothesized, that patients with a fast oral Tac clearance develop higher Tac peak levels than patients with a slow oral Tac clearance. Therefore, C0 and C2 Tac levels were determined in an additional cohort of 56 RTx patients. Additionally, we assessed C4 levels and the area under the curve (AUC) in 25 of these 56 individuals. For C0, 12 h trough levels were assessed. C2 was assessed 2 h and C4 4 h after intake of the morning dose, respectively. Whole blood was analyzed for Tac (automated tacrolimus (TACR) assay; Dimension Clinical Chemistry System; Siemens Healthcare Diagnostic GmbH; Eschborn; Germany). In addition, a cell culture model using supra-therapeutic Tac concentrations was used to mimic the different Tac profiles of patients with fast and slow oral Tac clearance (see below).

All patients received an induction therapy with basiliximab or anti T-lymphocyte antibody and an immunosuppressive regimen containing immediate release tacrolimus (Prograf©), mycophenolate (CellCept©/Myfortic©), and prednisolone (Soludecortin H© /Decortin H©). 

Patients’ demographics were taken from the clinical hospital database and are presented in Table 1, and Tables S1 and S2.

The study was performed in accordance with the Declaration of Helsinki and approved by the local ethics committee (Ethik Kommission der Ärztekammer Westfalen-Lippe und der Medizinischen Fakultät der Westfälischen Wilhelms-Universität, 2017-407-f-S). Prior to analysis, all patient data were anonymized. Written informed consent with regard to recording their clinical data was given by all participants at the time of transplantation or inclusion into the study. Recipients aged <18 years, pregnant women, or patients with uncontrolled infection, tumor, or hypertension were excluded from the study.

### 2.2. Assessment of Pulse Wave Velocity and Glycocalyx

Besides tubular changes tacrolimus toxicity comprises vascular effects (vasoconstriction, arteriolopathy) as well. After having linked tubular changes with the Tac oral clearance in patients and a cell culture model, respectively, we conducted a second prospective study to assess potential Tac metabolism-related vascular changes. Therefore, we measured pulse wave velocity (PVW) and the glycocalyx as surrogate parameters of endothelial dysfunction/arterial stiffness in 120 stable RTx outpatients (30 patients with a C/D ratio < 1.05 ng/mL×1/mg and 90 patients with a C/D ratio ≥ 1.05 ng/mL×1/mg). Arterial stiffness was assessed as pulse wave velocity (PWV) using cuff-based oscillometry (Mobil-O-Graph, IEM, Stolberg, Germany) [18,19]. Subjects rested for 10 min at 23 °C before the baseline hemodynamic measurements were performed. Initially, brachial systolic blood pressure (mmHg) was measured. Two sequential measurements separated by a 5-min interval were obtained. The mean PWV was used for the analysis only if the PWV difference between the assessments was <0.5 m/sec. Otherwise, a third measure was conducted and the median of all values was calculated as published before [20]. An experienced single operator performed the measurements.

**Figure 2:**
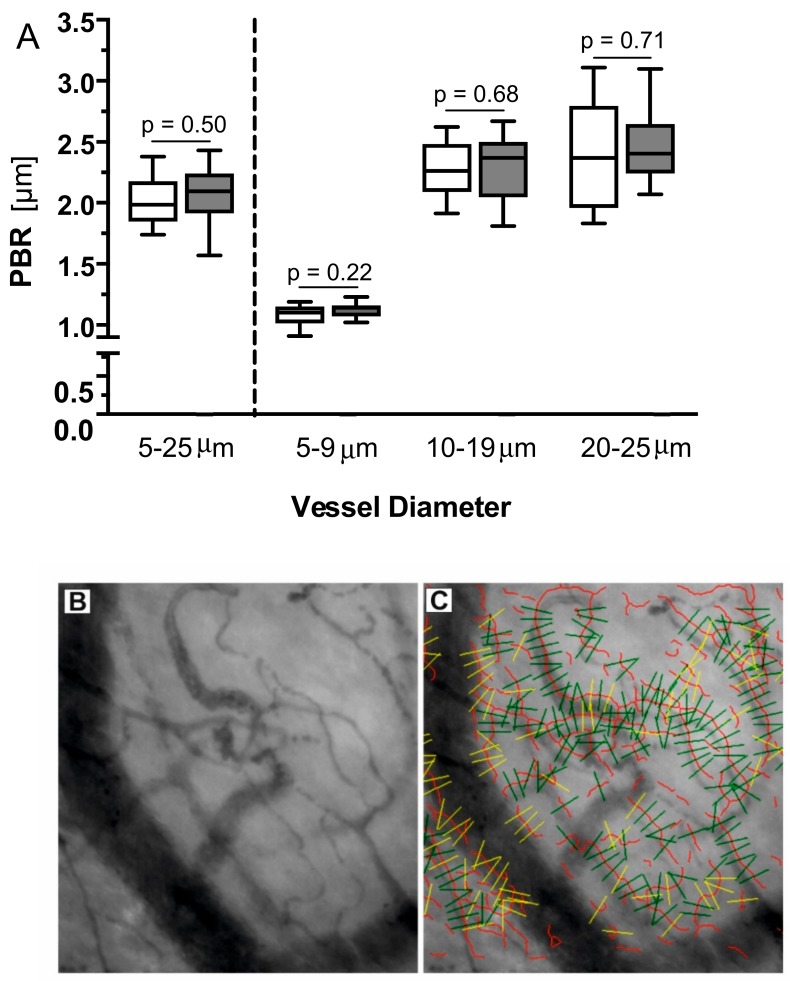
(**A**): Boxplots of PBR values of patients with a high C/D ratio (white) and low C/D ratio (grey) based on the different microvascular diameter ranges. No difference was detected between the groups. (**B**): Representative image of the sublingual mucosa acquired with the SDF camera in a kidney transplant patient. (**C**): Exemplary picture of a video recording showing the automatic identification of all available micro-vessels with a diameter between 5 to 25 μm. Vascular segments are marked every 10 μm along the assessed microvasculature (red lines) by the GlycoCheck software (green lines: valid segments for further analysis, yellow lines: discarded by quality check).

Furthermore, we prospectively assessed the dynamic lateral red blood cell movement into the glycocalyx that is expressed as the perfused boundary region (PBR) (in μm) in a subset of 28 (14 fast metabolizer) stable and matched RTx patients using bedside real-time intravital microscopy [21]. The sublingual microvasculature was visualized and examined with the use of GlycoCheckTM Software, coupled to a sidestream dark field (SDF) camera (CapiScope HVCS, KK Technology, Honiton, UK) by an experienced single operator, as thoroughly described before [21]. Briefly, the SDF camera uses stroboscopic diodes (540 nm) to detect the hemoglobin of the red blood cells (RBC). The GlycoCheck software allows automatic video recording when predefined image quality criteria (motion, intensity, focus) are met. The software automatically identifies all available micro-vessels with a diameter between 5 to 25 μm and marks vascular segments every 10 μm along the assessed microvasculature. Before further analysis of the videos, it performs an automatic quality check (Figure 2C). Invalid vascular segments are marked with yellow and automatically discarded, while all valid vascular segments (green lines) are subjected to further analysis. The software measures the PBR (in μm); an inverse parameter of glycocalyx dimensions. Specifically, the dynamic lateral RBC movement towards the endothelial wall is assessed in an average of about 3000 different vascular segments with a diameter from 5 to 25 μm (Figure 2B,C). An impaired endothelial glycocalyx allows RBCs to penetrate more deeply towards the endothelium, which translates into higher PBR values.

### 2.3. Cell Culture

Tubular epithelial cells (NRK-52E; ATCC) were cultivated in Dulbecco’s modified Eagle medium (DMEM) (Invitrogen, Darmstadt, Germany) supplemented with 10% fetal calf serum (FCS) (Biochrom, Berlin, Germany), 1% antibiotics (Pen/Strep) and L-Glutamine (PAA; Cölbe, Germany), and were cultured at 37 °C and 5% CO_2_. NRK-52E cells were grown in 12-well or 96-well plates until 80% confluence followed by treatment with tacrolimus (Prograf® i.v., Astellas, Munich, Germany) diluted in 0.9% sodium chloride) or medium only as a control over 12 h. Tac working solutions were freshly prepared by appropriate dilution of stock solution in the culture medium. Due to the inherent robustness of rat cells, titration series were conducted to determine the optimal tacrolimus concentrations that induce an appropriate reduction in cell viability. Culture medium was changed every hour using the indicated Tac concentrations between 6 and 20 µg/mL that were based on our titration studies and previous studies by Lamoureux et al. (Figure S1) [22]. After the incubation period of 12 h, the culture medium was removed and cells were washed three times with PBS and then prepared for quantitative Western blot analysis or MTT assay as described below. All samples were tested in triplicate wells. Data are representative of three different experiments.

### 2.4. Lysate Preparation and Western Blot Analysis

As fibroblast growth factor-inducible 14 (Fn14) is involved in the pathogenesis of CNIT we analyzed its expression in Tac-treated NRK cells using primary antibodies against α-actinin 4 and Fn14, respectively [3]. 

Preparation and quantitative Western blot analysis of cell lysates have been described previously [23]. Briefly, for quantitative Western blotting, cells were grown on dishes and then scraped into 1x LaemmLi (4% SDS, 5% 2-mercaptoethanol, 10% glycerol, 0.002% bromophenol blue, 0.0625 M Tris-HCl; pH 6.8). Samples were shaken at 1000 rpm for 2 h and then subjected to ultrasound bath treatment for 15 min. After being boiled for 5 min, equal volumes of cell lysates were separated onto 10% SDS-PAGE gels (Bio-Rad). Proteins were transferred to a PVDF membrane (Millipore) and incubated for 1 h at room temperature in blocking buffer (5% skim milk powder dissolved in TBS containing 0.05% Tween-20). A primary antibody against Fn14 (Cell Signaling, Danvers, MA, USA) was used in a 1:1000 dilution in TBS–Tween-20 and incubated at 4 °C overnight. After being washed three times with TBS–Tween-20, the membrane was incubated with horseradish peroxidase–coupled secondary antibodies (Jackson Immunoresearch, via Dianova, Hamburg, Germany) diluted 1:5000 in blocking buffer for 45 min at room temperature. After three washes, the Western blot was developed using a chemiluminescence detection reagent (Roche). For normalization of band density following chemiluminescence detection the samples were equalized using α-actinin (Enzo, Loerrach, Germany) as the loading control. All samples were tested in triplicate wells and three different experiments.

### 2.5. MTT Test

Cell viability was assessed by a colorimetric assay, which is based on the conversion of dissolved yellow 3-[4,5-dimethylthiazol-2-yl]-2,5-diphenyltetrazolium bromide (MTT) to insoluble purple formazan by cleavage of the tetrazolium ring by mitochondrial dehydrogenases of living cells as previously described [24,25]. Therefore, this MTT assay offers precise quantification of cell viability in mammalian cell cultures. Briefly, after Tac treatment for 12 h, the medium was carefully removed and replaced by 200 μL of fresh complete cell culture medium. 10 μL of MTT solution containing 5 mg/mL of the dye were added to each well, and the cells were again incubated for 3 h. The medium was then removed and 100 μL of lysis buffer containing 10% (w/v) sodium dodecyl sulfate and 40% (v/v) dimethylformamide was added to each well. The plates were shaken for 10 min to destroy the cell structure and dissolve the blue formazan dye. Finally, the absorbance was measured at 590 nm using an automated microtiter plate reader (Infinite M200; Tecan, Männedorf, Switzerland). The percentage of viable cells in the untreated controls was compared to that for the respective Tac treatments.

### 2.6. Statistical Analysis

Statistical analysis were performed using IBM SPSS® Statistics 25 for Windows (IBM Corporation, Somers, NY, USA) or GraphPad Prism version 4.0 (GraphPad Sofware, La Jolla, CA, USA). Normally distributed continuous variables are shown as mean ± standard deviation (SD) or as mean ± SEM and non-normally distributed continuous variables as median and first and third quartiles (interquartile range, IQR). Absolute and relative frequencies have been given for categorical variables. Pairs of independent groups were compared using the Student’s t-test for normally distributed data, Mann–Whitney U test for non-normal data, and Fisher’s exact test for categorical variables. To compare paired data, we used the Wilcoxon test for continuous variables and the McNemar test for categorical variables. Comparison among groups in Western blot experiments was performed by one-way ANOVA along with post-hoc Tukey test. *p*-values < 0.05 were considered as statistically noticeable.

## 3. Results

### 3.1. Histology

**Figure 3:**
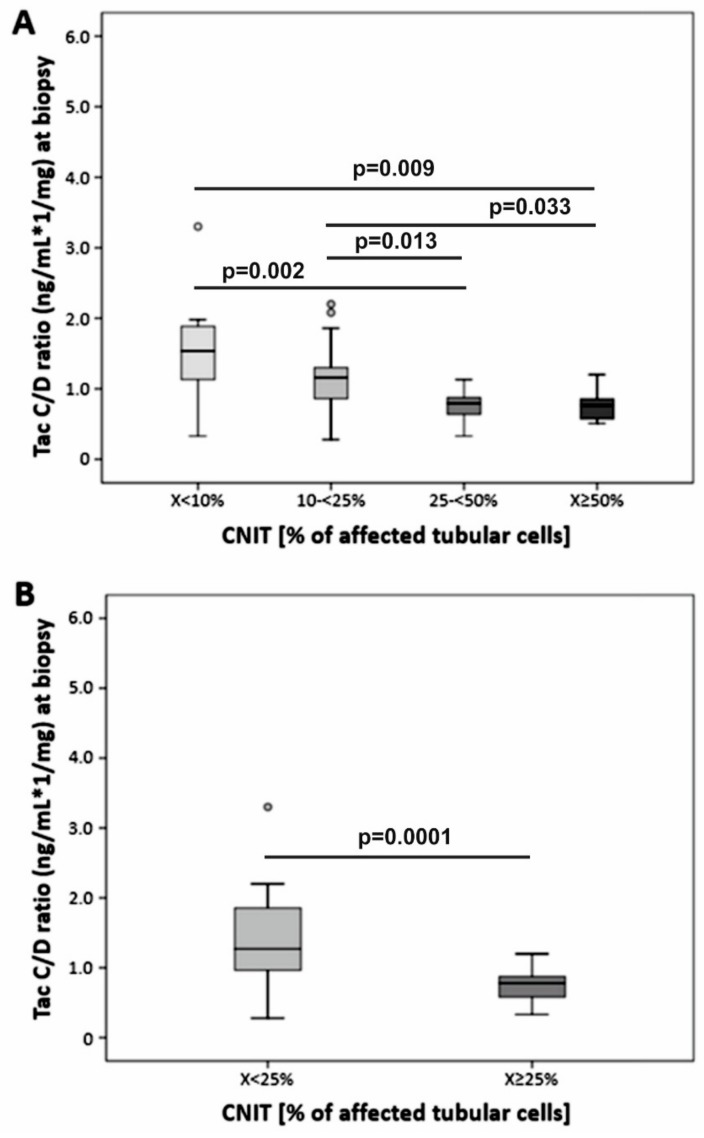
Histological analysis of calcineurin inhibitor-induced nephrotoxicity (CNIT) in kidney transplant biopsies, assessing the degree percentage of tubular cells with isometric vacuolization of the cytoplasm. The C/D ratio indicated a strong negative association with the severity of CNIT.

The histological re-analysis of 55 consecutive kidney transplant biopsy samples from patients (low C/D ratio, *n* = 27) with evidence of CNIT indicated by the presence of the characteristic isometric vacuolization of the tubular epithelial cells in < 10% (*n* = 20), 10–24% (*n* = 15), 25–49% (*n* = 12) and eight biopsies ≥ 50% of affected tubular cells. For further comparison, samples were regrouped according to < 25% (*n* = 35) or ≥ 25% (*n* = 20) tubular isometric vacuolization (Figure 3, Table 1). 

**Figure 4:**
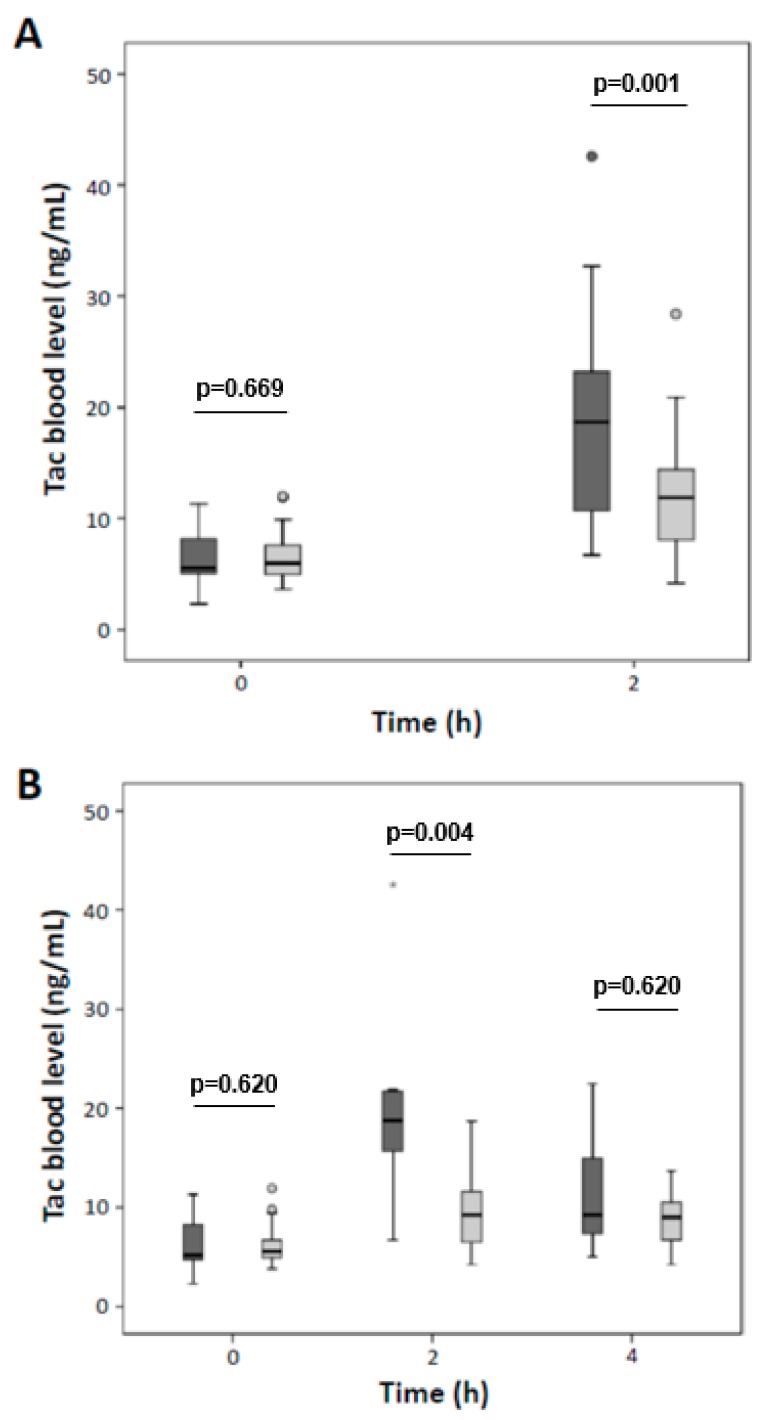
Presented are C0, C2 (*n* = 56) (**A**), and C4 (*n* = 25) (**B**) Tac levels in stable kidney transplanted patients. While the trough level (C0) and the C4 level were comparable between patients with a high (dark grey bars) and low (light grey bars) C/D ratio, C2 levels were significantly increased in patients with a low C/D ratio.

Although the trough levels at the time of biopsy were similar for both groups (Table 2), the degree of CNIT indicated a strong negative association to the C/D ratio values (Figure 3). Trough levels in 56 additional patients were comparable between patients with a low and high C/D ratio (6.3 ± 2.4 µg/L vs. 6.6 ± 2.2 µg/L respectively; *p* = 0.669). However, patients with a low C/D ratio displayed significantly higher C2 levels (19.2 ± 8.7 µg/L vs. 12.2 ± 5.2 µg/L, respectively; *p* = 0.001, Figure 4A). In a subgroup of 25 patients, C0 levels (6.3 ± 3.2 µg/L vs. 6.2 ± 2.3 µg/L, respectively; *p* = 0.620) and C4 (11.3 ± 5.8 µg/L vs. 9.0 ± 2.7 µg/L, respectively; *p* = 0.466) were comparable between groups. However, C2 levels of patients with a low C/D ratio were increased (20.2 ± 10.3 µg/L vs. 9.8 ± 4.2 µg/L, respectively; *p* = 0.004, Figure 4B). 

### 3.2. Pulse Wave Velocity Analysis

**Figure 5:**
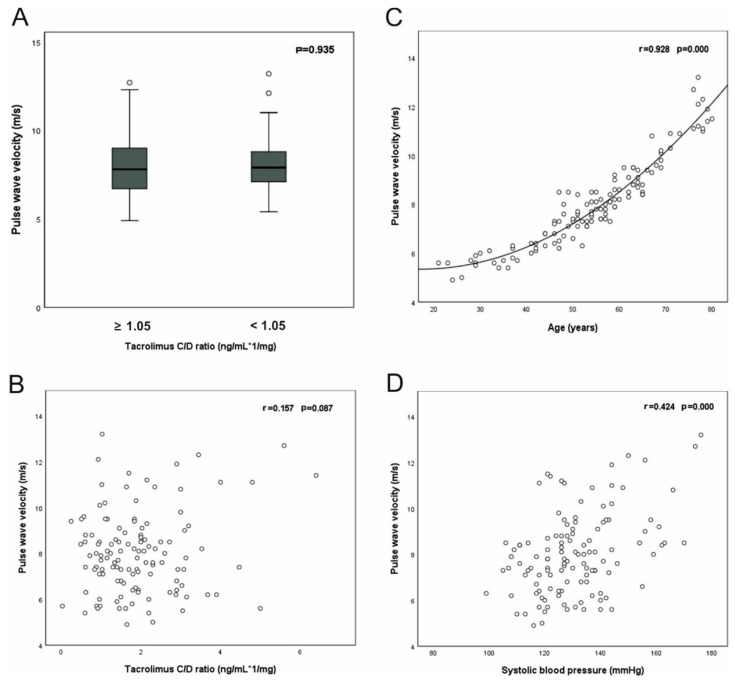
Pulse wave analysis of kidney transplanted patients. No correlation was observed between the C/D ratio and pulse wave velocity (**A**,**B**). The scatter plot in (**C**) indicates a strong quadratic relation of age and pulse wave velocity. Systolic blood pressure showed a moderately strong correlation to pulse wave velocity (**D**). R: Pearson correlation coefficient.

PWV correlated with age and systolic blood pressure (SBP) but not with the C/D ratio (Table S1, Figure 5).

### 3.3. Glycocalyx Analysis

The PBR, an inverse parameter of endothelial glycocalyx dimensions in sublingual vessels, was comparable between the patients with a low and a high C/D ratio (Figure 2).

### 3.4. Cell Culture

**Figure 6:**
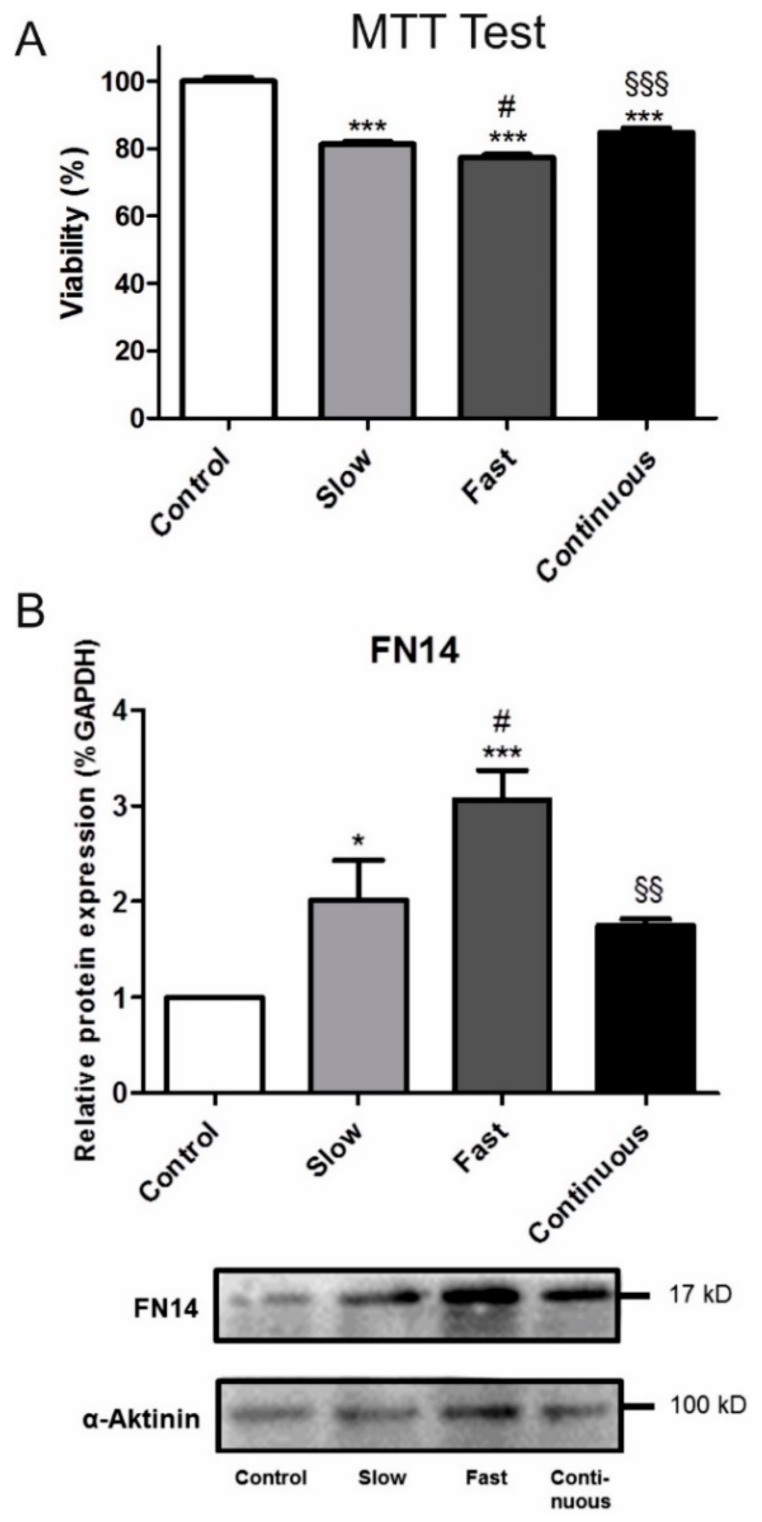
The viability of tubular epithelial cells (NRK-52E; ATCC) assessed by 3-[4,5-dimethylthiazol-2-yl]-2,5-diphenyltetrazolium bromide (MTT) test (**A**). Cells treated for 12 h with Tac according to the pharmakokinetic profile of fast metabolizers (Fast) showed the most reduced viability (*** *p* < 0.001 vs. Control, # *p* < 0.05 vs. Tac slow, ^§§§^ *p* < 0.001 vs. Tac fast. Western blot analysis of Fn14 expression in NRK cells (**B**), showed higher Fn14 expression levels in NRK cells when compared to the other groups (* *p* < 0.05 vs. Control, *** *p* < 0.001 vs. Control, # *p* < 0.05 vs. Tac slow. ^§§^ *p* < 0.01 vs. Tac fast). An exemplary Western blot is presented below.

Titration series revealed a tacrolimus concentration between 6 µg/mL and 20 µg/mL to induce an appropriate reduction of NRK cell viability in MTT assays. Tac exposure decreased the viability of NRK cells (Figure 6A) the most in cells that were treated with Tac corresponding to the PK profiles of fast metabolizers (6 µg/mL to 19 µg/mL) (77.3%) compared to cells that were exposed to continuous Tac treatment (8.5 µg/mL) or to Tac corresponding to the PK profiles of slow metabolizers (6 µg/mL to 12 µg/mL, respectively (81.3% vs. 84.7%, Figure 6A). Accordingly, these cells showed the highest Fn14 expression (Figure 6B).

## 4. Discussion

CNIT is a frequent complication of Tac exposure and is associated with reduced renal function and kidney graft loss. So far, no specific treatment of CNIT is available. Therefore, approaches to minimize its occurrence and identify the patients at risk are required. 

The C/D ratio is a simple estimate of the Tac metabolism rate and is therefore useful to stratify patients’ risk [1]. Since we identified a strong negative association between the C/D ratio and degree of acute CNIT observed in RTx biopsies, we describe in this study for the first time that a low C/D ratio (defined as a C/D ratio < 1.05 µg/L×1/mg) is linked to CNIT severity (Figure 3). 

We previously observed that a low C/D ratio is associated with an inferior renal function after transplantation [1]. This effect persisted in a five-year follow-up, and a low C/D ratio was identified as an independent risk factor for a decreased graft and patient survival [16]. In an earlier study, the indication biopsy rate, that histologically showed more frequently CNIT in patients with a low C/D ratio, was higher than in patients with a high C/D ratio [9]. However, the sample size in this study was low and no information was available on the severity of the CNIT lesions. To fill these gaps, we performed the presented combined retro- and prospective studies to further investigate the influence of fast Tac metabolism on CNIT occurrence and severity.

Despite higher daily Tac dosages, patients with a low C/D ratio do not usually display higher trough concentrations, AUCs, and Tac metabolites compared to patients with a high C/D ratio (Table 2) [5,6,7,9,26]. Patients’ PK profiles, including the peak level concentration, must consequently differ—a finding that we can confirm (Figure 4) [27]. In a study on the PK Tac profiles of stable RTx patients, Miura et al. presented in Figure 1 12 h PK profiles from their patients whose Tac concentration sharply increases to an early, high peak after Tac intake followed by a rapid decrease of the Tac blood level (suggestive of a low C/D ratio). In contrast, other patients exhibited a slow increase of Tac levels to a lower peak level after Tac intake that was followed by a slower decrease of Tac concentration to the trough level (suggestive of a high C/D ratio) [28]. In this regard, a randomized, prospective crossover study that assessed 24 h Tac PK profiles in genotyped, kidney transplanted African Americans provided informative insights [29]. African Americans, who are predominantly CYP3A5 expressors and therefore fast metabolizers, required double doses of weight-normalized immediate release (IR) Tac as compared to CYP3A5 non-expressors. Despite comparable Tac AUCs and a similar total exposure of fast and slow metabolizers to the compound, PK profiles and the exposure to Tac at different time points after intake differ. This is important because peak level concentrations that presumably cause temporary Tac overexposure are linked, e.g., to neurotoxicity [30]. 

In our cell culture model, the viability of NRK tubular epithelial cells significantly decreased when cells were incubated with Tac according to the PK profiles of fast metabolizers (Figure 6). These most affected cells notably expressed the highest amount of Fn14, a receptor protein known to be involved in the pathogenesis of CNIT [3]. It has to be considered that due to the robustness of NRK cells we applied Tac concentrations that are supra-therapeutic compared to Tac blood concentrations that are observed in patients and a direct translation into clinical practice and exact modelling of patients’ Tac exposure, which is much more complex and influenced by many factors in vivo, is limited. However, by using this rather simple in vitro system we herein provide insights into the pathophysiologic effects of the different Tac PK profiles. Since endothelial dysfunction is essentially involved in the pathogenesis of acute CNIT, we exemplarily analyzed the functioning of the glycocalyx and the PWV; such analysis has been recently shown to be of additive value for the assessment of vessel function [2,31]. However, no differences were observed between the matched patients with low and high C/D ratios. The underlying causes for this could be the small sample size or a relatively small impact of Tac on the vessels [19]. Tac, in contrast to cyclosporine, does not reduce renal plasma flow, GFR, or blood pressure—at least not in healthy subjects [32].

Our study has limitations. First, Tac metabolites have not been measured and the analysis of Tac peak levels was not performed in the same group of RTx patients in whom the association of C/D ratio and histological CNIT was investigated since this part of the study was of a retrospective nature and could therefore only be hypothesis generating. Assessment of Tac metabolism is complex as it includes different processes such as uptake, metabolism in the intestine, liver, blood, and kidneys as well as its elimination. All these steps underlie many influencing factors (e.g., genetics, albumin level, hematocrit, differences in absorption and compliance). As we did not assess Tac metabolites, the C/D ratio can only serve as an estimate (sum of all effects that affect Tac metabolism in vivo) of the true Tac metabolism rate. Rather, the C/D ratio constitutes a simple tool to describe the stable condition between uptake and elimination of Tac in the blood. Nevertheless, a correlation between the C/D ratio and several CYP3A subtypes has already been shown by others (e.g. Reference [33]). To note, in terms of clinical outcomes it has been very recently demonstrated by Jouve et al. that e.g., genetics, were in contrast to the C/D ratio not suitable to predict the outcome of patients [13]. Despite the aforementioned limitations, the C/D ratio can serve as a simple estimate of the metabolism rate which is practical, cost-effective and can assist physicians in the daily routine for risk assessment and to individualize their patients’ immunosuppressive therapy. Second, the vascular parameters PBR and PWV have not been analyzed directly in the kidney but are usually extrapolated from measures at other body sides. Therefore, local effects of Tac on the renal endothelial might have been missed using our approach. Moreover, there are further parameters that can impact on the glycocalyx and the vascular stiffness such as diabetes or hypertension (frequently present in RTx patients and also potentially related to Tac) which have not been investigated in our study. Third, the sample size of our single center study is limited.

Despite these limitations, we demonstrated that a low C/D ratio is associated with significantly higher Tac C2 levels and more severe CNIT. We also showed that systemic markers of endothelial (dys-)function were not associated with the C/D ratio and NRK tubular epithelial cells in vitro were most affected when exposed to Tac according to a fast metabolism PK profile. The C/D ratio may, therefore, be an appropriate tool for identifying patients at risk of developing CNIT.

## Figures and Tables

**Table 1:** Patient characteristic: Histological analysis.

CNI Nephrotoxicity	x < 25% (*n* = 35)	x ≥ 25% (*n* = 20)	*p*-Value
Age (years, mean ± SD)	57.8 ± 12.4	50.2 ± 20.2	0.014 ^a^
Male sex, *n* (%)	24 (68.6)	12 (60)	0.566 ^b^
BMI (kg/m^2^, mean ± SD)	25.5 ± 5.2	25.6 ± 5.3	0.981 ^a^
Prednisolone dose (mg, mean ± SD)	10.0 ± 6.3	14.9 ± 17.5	0.239 ^a^
Living donor transplantation, *n* (%)	26 (74.3)	14 (70)	0.761 ^b^
ESP, *n* (%)	9 (25.7)	1 (5)	0.075 ^b^
Combined RTx + liver Tx, *n* (%)	3 (8.6)	1 (5)	1 ^b^
Previous Tx, *n* (%)	3 (8.6)	0	0.293 ^b^
ABOi, *n* (%)	4 (11.4)	2 (10)	1 ^b^
CIT (hours, mean ± SD)	9.2 ± 5.0	8.5 ± 5.0	0.669 ^a^
WIT (min, mean ± SD)	32.5 ± 8.1	32.5 ± 5.4	0.418 ^a^
DGF	11 (31.4)	2 (10)	0.107 ^b^
Donor data			
Male donor sex, *n* (%)	13 (47,1)	14 (70)	0.026 ^b^
Donor age (years, mean ± SD)	61.1 ± 15.7	52.2±14.7	0.073 ^a^
Time from RTx to biopsy (days)	63 (3–2877)	223 (10–5057)	0.059 ^c^

Patients with a CNI nephrotoxicity < 25% were observed to be older and received more female allografts; CNI, calcineurin inhibitor; BMI, body mass index; ESP, European senior program; RTx, renal transplantation, Tx, transplantation; ABOi, ABO incompatible transplantation; CIT, cold ischemia time, WIT, warm ischemia time, DGF, delayed graft function; ^a^ Student’s *t*-test; ^b^ Fisher’s exact test; ^c^ Mann–Whitney U test.

**Table 2:** Tac trough level and dose of two calcineurin inhibitor toxicity groups.

	x < 25% (*n* = 35)	x ≥ 25% (*n* = 20)	*p*-Value
Tac trough level (ng/mL ± SD)	6.0 (3.1–15.1)	5.8 (2.4–12.5)	0.431
Tac dose (mg, mean ± SD)	5.0 (1.0–18.0)	8.0 (3.0–16.0)	0.009
C/D ratio, ng/mL×1/mg, median (min-max)	1.27 (0.28–5.03)	0.78 (0.33–1.20)	< 0.001

Tac, tacrolimus; Mann–Whitney U test.

## Supplementary Materials

The following are available online at https://www.mdpi.com/2077-0383/8/10/1586/s1. Figure S1: PK profiles used for NRK cell incubation, Table S1: Patient characteristics: Pulse wave analysis, Table S2: Patient demographics: Glycocalyx assessment.
