# Supplementary material for: A Low Tacrolimus Concentration/Dose Ratio Increases the Risk for the Development of Acute Calcineurin Inhibitor-Induced Nephrotoxicity
Source: J Clin Med. 2019 Oct 2;8(10):1586. doi: 10.3390/jcm8101586 (PMC6832469; doi:10.3390/jcm8101586)
Supplement: Supplementary file 1 [file jcm-08-01586-s001.pdf]

Journal of
Clinical Medicine

Supplemental Material

Table S1. Patient characteristics: Pulse wave analysis.

	slow metabolizers (*n* = 90)	fast metabolizers (*n* = 30)	*p*-value
**Age (years, mean ± SD)**	54.3 ± 13.6	54.1 ± 14.2	0.936 [a]
**Male sex, *n* (%)**	55 (61.1)	16 (53.3)	0.522 [c]
**BMI (kg/m^2, mean ± SD)**	26.8 ± 4.2	26.4 ± 4.4	0.627 [a]
**Pre-existing recipient hypertension, n (%)**	81 (90.0)	28 (93.3)	0.729 [c]
**Diagnosis of ESRD, n (%)**			
Hypertension	7 (7.8)	1 (3.3)	
Diabetes	11 (12.2)	3 (10.0)	
Polycystic kidney disease	15 (16.7)	3 (10.0)	
Obstructive nephropathy	4 (4.4)	2 (6.7)	
Glomerulonephritis	23 (25.6)	7 (23.3)	0.596 [d]
FSGS	4 (4.4)	(0.0)	
Interstitial nephritis	7 (7.8)	4 (13.3)	
Other	14 (15.6)	9 (30.0)	
Unknown	5 (5.6)	1 (3.3)	
**Dialysis vintage (months, median (IQR))**	41.5 (59)	65.0 (96)	0.154 [a]
**≥ 1 prior kidney transplant, *n* (%)**	10 (11.1)	4 (13.3)	0.570 [c]
**Living donor transplantation**	34 (37.8)	9 (30.0)	0.514 [c]
**Number HLA mismatch, *n* (%)**			
0–3	29 (32.2)	11 (36.7)	0.661 [c]
4–6	61 (67.8)	19 (63.3)	
**Current PRA, *n* (%)**			
0–20 %	85 (94.4)	26 (86.7)	0.225 [c]
> 20 %	5 (5.6)	4 (13.3)	
**Cold ischemia time (hours, mean ± SD)**	8.1 ± 5.7	8.9 ± 5.3	0.553 [a]
**Warm ischemia time (min, mean ± SD)**	34.3 ± 6.9	35.2 ± 10.7	0.698 [a]
**Donor age (years, mean ± SD)**	49.4 ± 13.2	51.0 ± 13.9	0.575 [a]
**Donor male sex, n (%)**	40 (44.4)	20 (66.7)	0.057 [c]
**Recipient eGFR at 1yr (mL/min per 1.73 m^2, mean ± SD)**	56.8 ± 19.8	48.6 ± 16.9	0.070 [a]
**Recipient eGFR at pulse wave analysis (mL/min per 1.73 m^2, mean ± SD)**	49.9 ± 14.2	42.8 ± 15.8	0.024 [a]
**Time from Tx to Measurement (years, median, (1st, 3rd quartile))**	4.5 (3.0, 6.0)	4.6 (1.0, 6.7)	0.913 [b]
**Peripheral systolic blood pressure (mmHg, mean ± SD)**	130 ± 15	133 ± 17	0.438 [a]
**Peripheral diastolic blood pressure (mmHg, mean ± SD)**	85 ± 10	88 ± 13	0.213 [a]
**Mean arterial pressure (mmHg, mean ± SD)**	106 ± 11	108 ± 14	0.377 [a]

**Central systolic blood pressure (mmHg, mean ± SD)**	120 ± 14	124 ± 16	0.283 [a]
**Central diastolic blood pressure (mmHg, mean ± SD)**	86 ± 10	88 ± 14	0.398 [a]
**Pulse wave velocity (m/s, mean ± SD)**	8.0 ± 1.8	8.1 ± 1.9	0.935 [a]
**Augmentation index (mean ± SD)**	19.3 ± 14.6	14.8 ± 13.0	0.141 [a]
**Total vascular resistance (s*mmHg/mL)**	1.3 ± 0.2	1.3 ± 0.2	0.737 [a]

[a] Student's *t*-test, [b] Mann-Whitney U test, [c] Fisher's exact test, [d] Pearson's chi-squared test.

Table S2. Patient demographics: Glycocalyx assessment.

	slow metabolizers (*n* = 14)	fast metabolizers (*n* = 14)	*p*-value
**Age (years, mean ± SD)**	50.7 ± 13.3	48.7 ± 9.7	0.659[a]
**Male sex, n (%)**	6 (42.9)	7 (50.0)	1.000[c]
**BMI (kg/m^2, mean ± SD)**	23.6 ± 3.5	25.7 ± 5.8	0.272[a]
**Pre-existing recipient hypertension, n (%)**	13 (92.9)	12 (85.7)	1.000[c]
**Diagnosis of ESRD, n (%)**			
Hypertension	1 (7.1)	2 (14.3)	
Diabetes	1 (7.1)	0 (0.0)	
Polycystic kidney disease	1 (7.1)	0 (0.0)	
Obstructive nephropathy	0 (0.0)	1 (7.1)	
Glomerulonephritis	5 (35.7)	4 (28.6)	0.793[d]
FSGS	1 (7.1)	1 (7.1)	
Interstitial nephritis	3 (21.4)	1 (7.1)	
Other	1 (7.1)	4 (28.6)	
Unknown	1 (7.1)	1 (7.1)	
**Dialysis vintage (months, median (IQR))**	56.5 ± 36.9	60.1 ± 37.0	0.808[a]
**≥ 1 prior kidney transplant, *n* (%)**	3 (21.4)	4 (28.6)	1.000[c]
**Living donor transplantation**	3 (21.4)	2 (14.3)	1.000[c]
**Number HLA mismatch, n (%)**			
0–3	9 (64.3)	10 (71.4)	1.000[c]
4–6	5 (35.7)	4 (28.6)	
**Current PRA, n (%)**			
0–20 %	11 (78.6)	12 (85.7)	1.000[c]
> 20 %	3 (21.4)	2 (14.3)	
**Cold ischemia time (hours, mean ± SD)**	8.3 ± 5.2	10.2 ± 4.7	0.307[a]
**Warm ischemia time (min, mean ± SD)**	31.8 ± 6.9	32.2 ± 8.0	0.684[a]
**Donor age (years, mean ± SD)**	53.8 ± 13.8	51.1 (9.1)	0.546[a]
**Donor male sex, n (%)**	6 (42.9)	7 (50.0)	1.000[c]
**Recipient eGFR at measurement (ml/min per 1.73 m^2, mean ± SD)**	51.5 ± 13.9	45.8 ± 14.2	0.292[a]
**Time from Tx to Measurement (years, (median, 1st, 3rd quartile))**	4.0 (1.9, 12.0)	5.1 (2.4, 7.6)	0.874[b]
**Tac blood trough concentration (ng/ml, mean ± SD)**	3.5 ± 1.4	8.1 ± 2.6	<0.001[a]
**Tac daily dose (mg, mean ± SD)**	6.6 ± 2.3	6.1 ± 1.3	0.495[a]
**Tac C/D ratio (ng/mL × 1/mg, mean ± SD)**	2.1 ± 0.8	0.8 ± 0.2	<0.001[a]

[a]Student's *t*-test, [b]Mann-Whitney U test, [c]Fisher's exact test, [d]Pearson's chi-squared test.

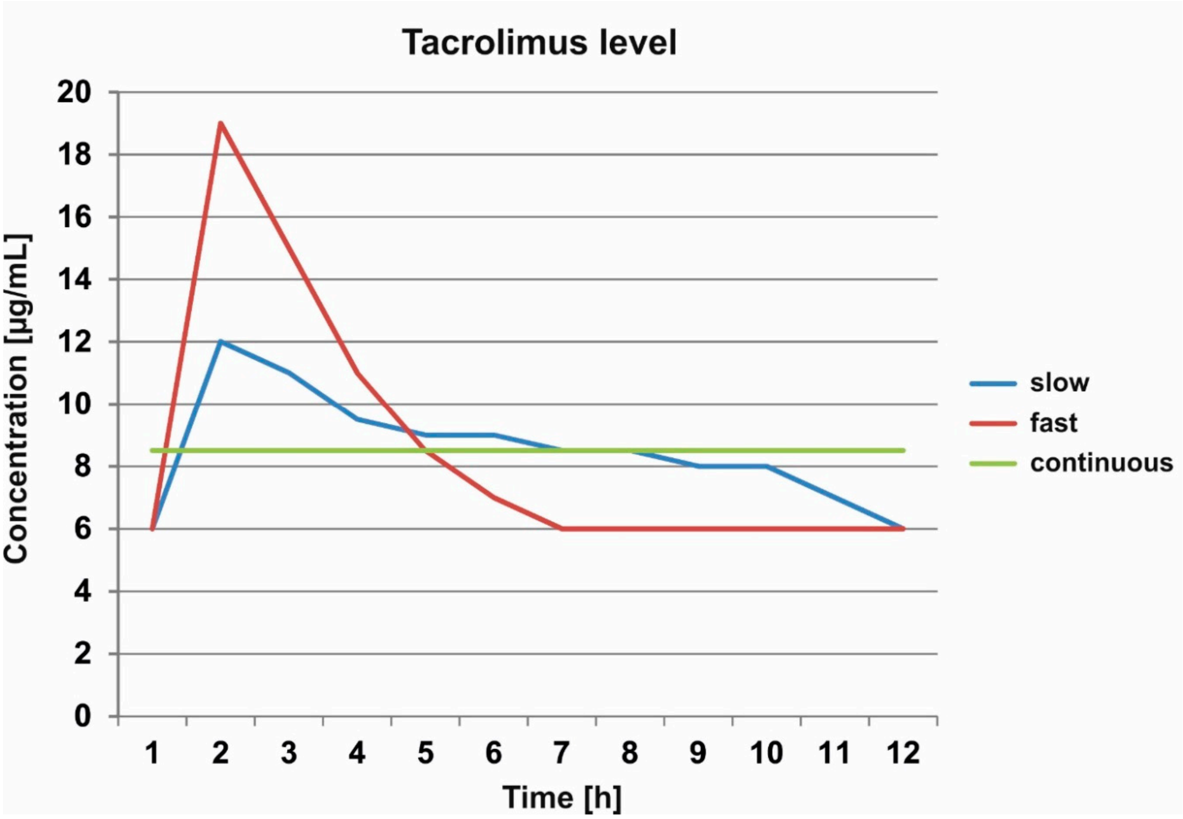

Figure S1. Pharmakokinetic profiles used for NRK cell incubation. Culture medium was changed every hour using the indicated Tac concentrations.
